# Heritable base-editing in *Arabidopsis* using RNA viral vectors

**DOI:** 10.1093/plphys/kiac206

**Published:** 2022-05-05

**Authors:** Degao Liu, Shuya Xuan, Lynn E Prichard, Lilee I Donahue, Changtian Pan, Ugrappa Nagalakshmi, Evan E Ellison, Colby G Starker, Savithramma P Dinesh-Kumar, Yiping Qi, Daniel F Voytas

**Affiliations:** Department of Genetics, Cell Biology and Development, University of Minnesota, St. Paul, Minnesota 55108, USA; Center for Precision Plant Genomics, University of Minnesota, St. Paul, Minnesota 55108, USA; Department of Genetics, Cell Biology and Development, University of Minnesota, St. Paul, Minnesota 55108, USA; Department of Genetics, Cell Biology and Development, University of Minnesota, St. Paul, Minnesota 55108, USA; Center for Precision Plant Genomics, University of Minnesota, St. Paul, Minnesota 55108, USA; Department of Genetics, Cell Biology and Development, University of Minnesota, St. Paul, Minnesota 55108, USA; Center for Precision Plant Genomics, University of Minnesota, St. Paul, Minnesota 55108, USA; Department of Plant Science and Landscape Architecture, University of Maryland, College Park, Maryland 20742, USA; Department of Plant Biology and The Genome Center, College of Biological Sciences, University of California, Davis, Davis, California 95616, USA; Department of Genetics, Cell Biology and Development, University of Minnesota, St. Paul, Minnesota 55108, USA; Center for Precision Plant Genomics, University of Minnesota, St. Paul, Minnesota 55108, USA; Department of Genetics, Cell Biology and Development, University of Minnesota, St. Paul, Minnesota 55108, USA; Center for Precision Plant Genomics, University of Minnesota, St. Paul, Minnesota 55108, USA; Department of Plant Biology and The Genome Center, College of Biological Sciences, University of California, Davis, Davis, California 95616, USA; Department of Plant Science and Landscape Architecture, University of Maryland, College Park, Maryland 20742, USA; Department of Genetics, Cell Biology and Development, University of Minnesota, St. Paul, Minnesota 55108, USA; Center for Precision Plant Genomics, University of Minnesota, St. Paul, Minnesota 55108, USA

## Abstract

Heritable base-editing using a viral delivery system enables high-throughput functional analysis of genes in *Arabidopsis*.

Dear Editor,

Precise base-editing technologies have great potential for studying plant gene function and accelerating crop improvement (Zong et al., 2017; Kang et al., 2018). To date, however, heritable base-editing has been difficult to achieve in many plants, including *Arabidopsis* (*Arabidopsis thaliana*). Transgenic *Arabidopsis* plants expressing base-editing reagents are often somatic mosaics in the first generation, and multiple generations are required to fix edited alleles (Kang et al., 2018; Li et al., 2019; Choi et al., 2021). Recently, heritable targeted mutagenesis has been achieved using RNA or DNA viruses to deliver single guide RNAs (sgRNAs) to the germline of Cas9-expressing transgenic plants (Ellison et al., 2020; Lei et al., 2021; Li et al., 2021; Nagalakshmi et al., 2022). Here, we show that this approach can be applied to base-editing using RNA viral vectors to deliver sgRNAs to transgenic *Arabidopsis* lines expressing a cytidine deaminase base-editor (Supplemental Figure S1, A and B). The base-editor, driven by the *Arabidopsis UBIQUITIN 10* (*UBI10*) promoter, is a Cas9 nickase fused to the human APOBEC3A (hA3A)/Y130F cytidine deaminase (Ren et al., 2021). Progeny of infected plants with heterozygous mutations in *PHYTOENE DESATURASE 3* (*PDS3*) and *CLOROPLASTOS ALTERADOS 1* (*CLA1*) were recovered at frequencies of 7.66% and 2.73%, respectively. Up to 8.90% of progeny from one plant were homozygous for mutations (C to T) in *PDS3* and exhibited an albino phenotype caused by introduction of a premature stop codon. Plants with gain-of-function mutations that confer herbicide tolerance (both homozygous and heterozygous) were recovered in *CELLULOSE SYNTHASE 3* (*CESA3*) at a frequency of 2.28%. These results suggest that virus-mediated base-editing can be performed at scale in *Arabidopsis*, allowing high-throughput functional analysis of genes through precise mutagenesis.

For our experiments, we identified two transgenic *Arabidopsis* lines (L4 and L8) that highly express the APOBEC3A-derived C-to-T base-editor, using reverse transcription quantitative PCR (RT-qPCR) (Supplemental Figure S1C and Supplemental Materials and Methods). Enhanced sgRNAs (esgRNA) targeting *PDS3* and *CLA1* were designed with a modified scaffold that increases their stability and promotes interactions with Cas9 (Li et al., 2020). Mutagenesis using these esgRNAs would convert a CAG codon of *PDS3* and a CGA of *CLA1* to premature TAG and TGA stop codons, respectively (Figure 1, A and B). Disruption of *PDS3* function results in a photobleached phenotype due to impaired carotenoid biosynthesis (Qin et al., 2007). *CLA1* is required for chloroplast development; homozygous knockout mutant plants have an albino phenotype (Estévez et al., 2000). Prior to cloning into *Tobacco Rattle Virus* (TRV) vectors, the esgRNAs were fused at their 3′-ends to a tRNA isoleucine (tRNA^Ileu^). We and others have previously shown that tRNAs and other RNAs that promote cell-to-cell movement can increase virus-mediated mutagenesis when fused to sgRNAs (Ellison et al., 2020; Lei et al., 2021; Nagalakshmi et al., 2022). The TRV vectors were delivered to the transgenic L8 line via the *Agrobacterium tumefaciens* flooding method (Nagalakshmi et al., 2022). The infected plants were grown under a 24°C/12-h-light and 22°C/12-h-dark cycle. Weak knockout phenotypes of *PDS3* and *CLA1* were observed in rosette leaves (Supplemental Figure S2). Base-editing was detected in rosette leaves, but not in cauline leaves. These results suggested that the base-editor activity was low and that base-edits were not likely fixed in the meristem at an early developmental stage.

**Figure 1:**
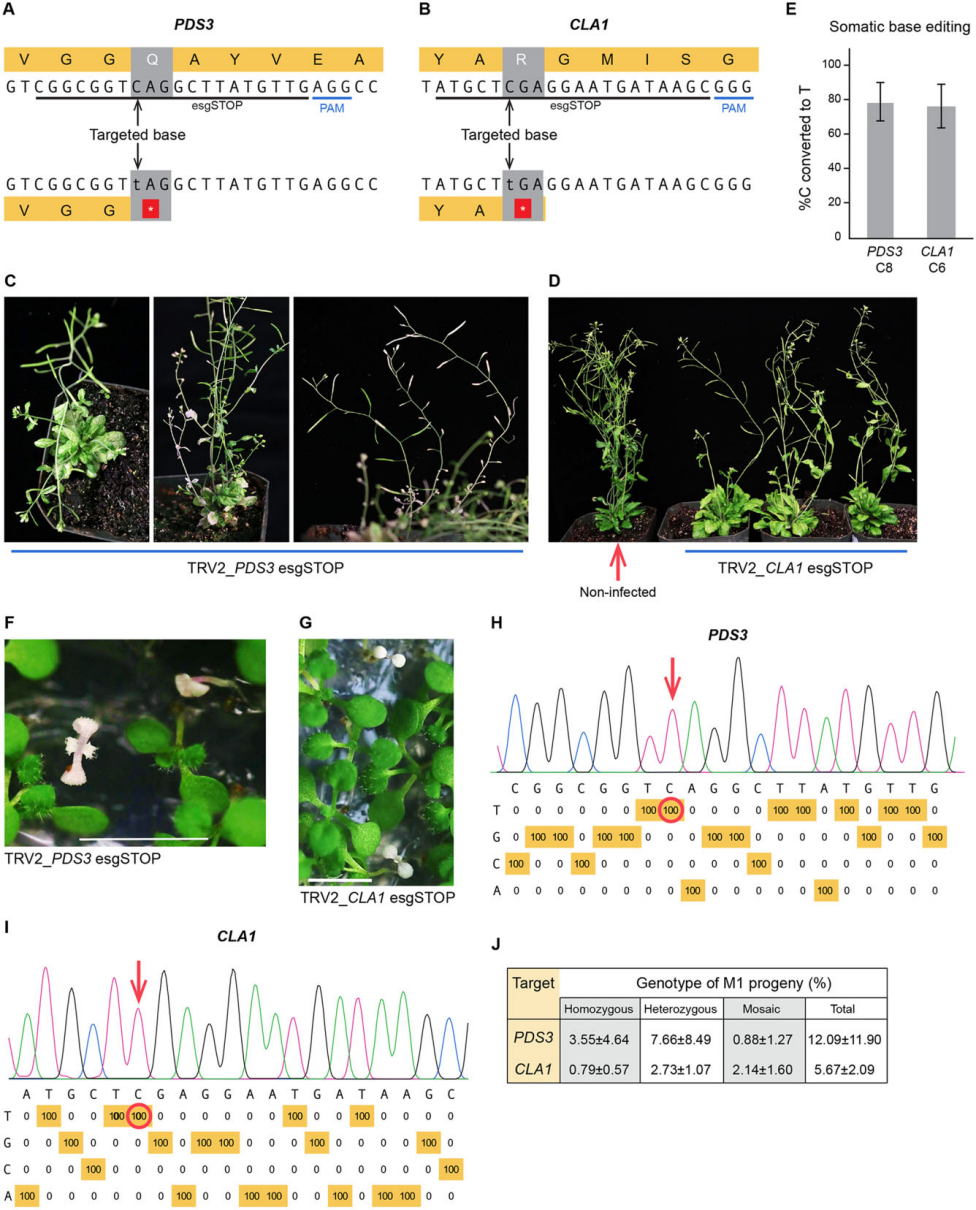
Heritable base-editing in *Arabidopsis* to create loss-of-function mutations at *PDS3* and *CLA1*. A, The esgRNA target in *PHYTOENE DESATURASE 3* (*PDS3*). Cytosine base editing precisely converts a CAG codon to a premature TAG stop codon. PAM: protospacer adjacent motif. B, The esgRNA target in *CLOROPLASTOS ALTERADOS 1* (*CLA1*). Base-editing converts a CGA codon to a TGA stop codon within the protein-coding sequence. C, *PDS3* knockout phenotypes in the M0 generation*.* The *Arabidopsis* plants were infected with TRV expressing the esgRNA targeting *PDS3*. Infected plants were grown at a constant temperature of 18°C with a 16-h/8-h day/night cycle, a light intensity of 100 μmol m^−2^ s^−1^, and 50% humidity. D, *CLA1* knockout phenotypes in the M0 generation. Plants were grown under the same conditions as in panel C. E, Base-editing frequencies in cauline leaves of the M0 generation. Data are presented as the mean ± sd (*PDS3*, *n* = 5; *CLA1*, *n* = 7). F, Albino phenotypes of the *PDS3* base-edited M1 progeny from the parental plants infected with TRV2-*PDS3* esgRNA. All seeds were harvested from infected parental plants and germinated on 0.5 MS medium containing 1% (w/v) sucrose. Scale bar: 0.5 cm. G, Albino phenotypes of the *CLA1* base-edited M1 seedlings. Scale bar: 0.5 cm. H, Representative base-editing profile of *PDS3* homozygous mutant M1 progeny. C–T conversion frequencies at every nucleotide in the 20-bp protospacer were quantified with EditR (https://moriaritylab.shinyapps.io/editr_v10/). I, Representative base-editing profile of homozygous mutant *CLA1* M1 progeny. J, The frequency of heritable gene editing in M1 seedlings. All seeds were harvested from infected plants. All white seedlings and green seedlings with white or yellow sectors were genotyped. Green seedlings were randomly sampled and genotyped. Most of the mutations were C-to-T edits. The mutation in one sectored seedling from PDS3_P1 was a 14-bp deletion; the mutations in two white seedlings and one green seedling from PDS3_P2 were 4-bp deletions. Data are presented as the mean ± sd (*n* = 3 parental lines). Genotyping data details are shown in Supplemental Table S1.

When RNA viral vectors are used to express hairpin RNAs, the resulting virus-induced gene silencing (VIGS) typically lasts for about three weeks (Senthil‐Kumar and Mysore, 2011). Growing infected plants at low temperatures (18°C–20°C) and under long-day conditions (Burch-Smith et al., 2006) can enhance VIGS, allowing it to persist for more than 2 years (Senthil‐Kumar and Mysore, 2011). We reasoned that base-editing might be improved if low temperatures and long days enhanced TRV replication and persistence. Furthermore, base-editing might be more efficient in meristematic cells if low temperatures prolong the time required for cell division. Indeed, infection of T2 seedlings with TRV-*PDS3* esgRNA from both L4 and L8 grown at 18°C and 16-h light resulted in plants with pronounced bleached spots in rosette leaves (Supplemental Figure S3). As the plants matured, completely photobleached branches, cauline leaves, flowers, and seed pods were observed (Figure 1C). When the TRV-*CLA1* esgRNA was delivered to L4, a yellowish-green phenotype appeared in both rosette leaves and cauline leaves (Figure 1D). The knockout phenotypes of *PDS3* or *CLA1* were observed in ∼30% of infected L4 plants. Base-editing efficiencies of *PDS3* and *CLA1* in cauline leaves were 78.6% and 76.9%, respectively (Figure 1E and Supplemental Figure S4).

To test whether the base-edits observed in the M0 generation were heritable, all seeds were harvested from three individual plants that were infected with each of the TRV vectors and displayed knockout phenotypes. Homozygous mutant albino progeny were recovered from plants infected with TRV2-*PDS3* esgRNA and *CLA1* esgRNA vectors at frequencies of 3.55% and 0.79%, respectively (Figure 1, F–J and Supplemental Table S1). One parental line, *PDS3*_P3, produced 8.90% (21/236) albino progeny (Supplemental Table S1). Plants heterozygous at *PDS3* and *CLA1* were recovered at frequencies of 7.66% and 2.73%, respectively (Figure 1J and Supplemental Table S1). The green seedlings with white or yellow zones and the green seedlings with editing frequencies between 15.1% and 35.0% or 65.1% and 85.0% were defined as mosaics. We observed *PDS3* and *CLA1* mosaics at a frequency of 0.88% and 2.14%, respectively (Figure 1J; Supplemental Figure S5 and Supplemental Table S1).

In addition to creating knockout mutations, base-editing is also a powerful tool for installing gain-of-function point mutations. We targeted a C-to-T transition mutation in *CESA3* that creates a S983F amino acid substitution conferring tolerance to the cellulose biosynthesis-inhibiting chemical compound C17 (5-(4-chlorophenyl)-7-(2-methoxyphenyl)-1,5,6,7-tetrahydro-[1,2,4]triazolo[1,5-a] pyrimidine) (Hu et al., 2019; Figure 2A). In a prior study, the floral dip method was used with base-editor BE3 driven by the constitutive *UBIQUITIN 4-2* promoter from parsley (*Petroselinum crispum*) and the *CESA3* sgRNA to create C17 tolerance; however, no homozygous mutants were recovered in approximately 2,000 T1 seedlings tested. Homozygous base-editing events were recovered in subsequent generations (Hu et al., 2019). Here, we delivered the TRV vector expressing the *CESA3* esgRNA to the L4 line. Base-editing frequencies of cauline leaves were 94.5% ± 9.11% (mean ± sd, *n* = 4) in the M0 plants. Seeds from infected parental plants were collected and directly sown on 0.5 MS medium supplemented with 1 μM C17 according to the method of Hu et al. (2019). C17-tolerant plants were observed and base-editing was confirmed (Figure 2, B–D). The frequency of homozygous, heterozygous, and mosaic plants were 1.83%, 0.45%, and 0.38%, respectively (Figure 2C and Supplemental Table S2). C-to-G editing was observed in two progeny from one parental line, *CESA3*_P3 (Supplemental Table S2). Primers used in this study are provided in Supplemental Table S3. The high number of homozygous mutants recovered may be because they grow better on the C17 selection medium relative to heterozygous and mosaic plants. We would like to note that in a companion study, TRV was used to deliver a *PDS* sgRNA to transgenic *Arabidopsis* plants expressing Cas9 (Nagalakshmi et al., 2022). Mutagenesis was assessed in seeds produced at different times during development (early, middle, and late stages). Interestingly, very few of the late stage progeny were albino, whereas 30%–60% of the middle stage progeny were completely photobleached. In our study, all seeds were collected together and it will be interesting to determine if base-editing frequencies differ during development of the inflorescence, which could guide approaches to enrich for base-editing events.

**Figure 2:**
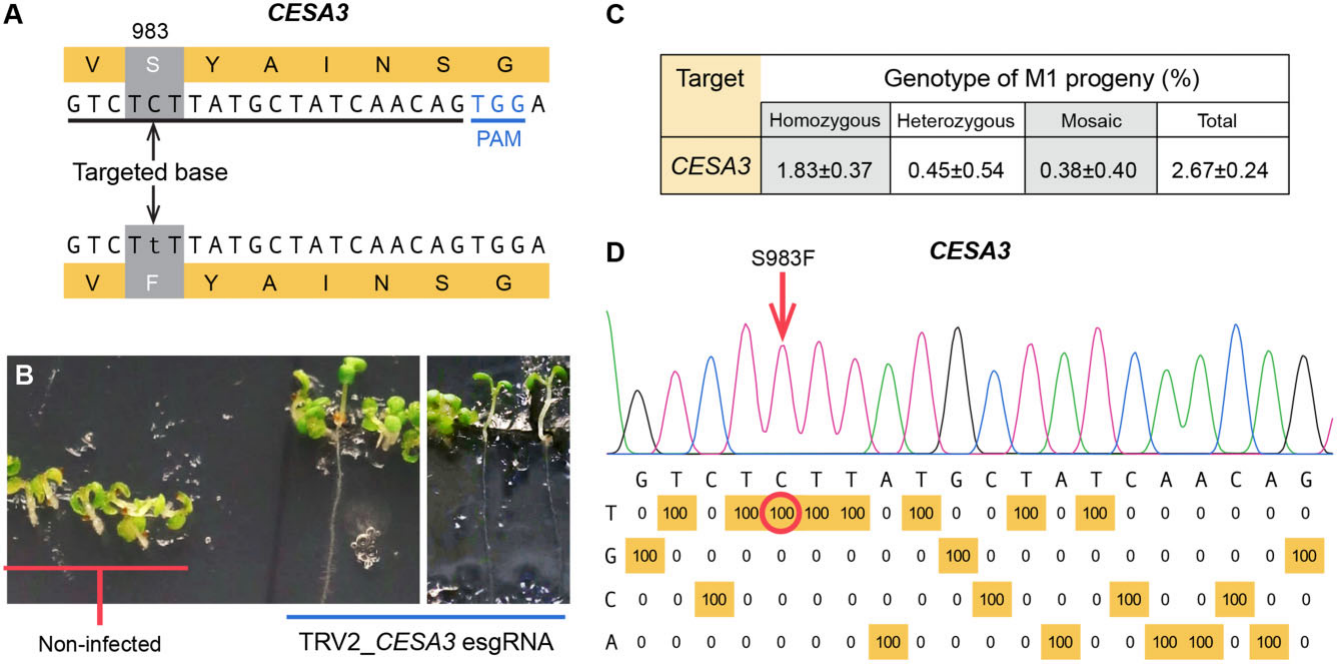
Heritable base-editing in *Arabidopsis* to create gain-of-function mutations at *CESA3.* A, The esgRNA target in *CELLULOSE SYNTHASE 3* (*CESA3*). A C-to-T transition induces an amino acid change (S983F) that confers tolerance to the cellulose biosynthesis-inhibiting chemical compound C17 (5-(4-chlorophenyl)-7-(2-methoxyphenyl)-1,5,6,7-tetrahydro-[1,2,4]triazolo[1,5-a] pyrimidine). PAM, protospacer adjacent motif. B, C17-tolerant *CESA3* base-edited M1 seedlings (5-d-old). Seeds from infected plants were harvested and germinated on 0.5 MS medium supplemented with 1% (w/v) sucrose and 1 μM C17. Root growth indicates tolerance to C17. C, The frequency of heritable base-editing in M1 seedlings. Data are presented as the mean ± SD (n = 3 parental lines). Genotyping data details are shown in Supplemental Table S2. D, Representative profile of CESA3 homozygous mutant M1 progeny. Base-editing frequencies at every nucleotide in the 20 bp protospacer were quantified with EditR (https://moriaritylab.shinyapps.io/editr_v10/).

Whereas the *Arabidopsis* floral dip transformation method has been previously used to deliver base-editing reagents, germline edits were difficult to attain. The recovery of homozygous mutants required screening progeny through several generations (Kang et al., 2018; Hu et al., 2019; Li et al., 2019). In contrast, our viral delivery method enables functional analysis of gene knockouts or point mutations in as early as the M0 generation. Further, homozygous mutants can be recovered in the M1 generation. An advantage of the floral dip method is that antibiotic selection can be used to enrich for T1 lines with active transgene expression. Because our TRV delivery system enables efficient multiplexed gene editing (Ellison et al., 2020; Nagalakshmi et al., 2022), we are developing a multiplexed base-editing approach to enable selection of plants that have undergone gene editing. We will use TRV to deliver multiple esgRNAs, including the *CESA3* esgRNA to create herbicide tolerance. Selection on the C17 herbicide will identify plants that have undergone base-editing at *CESA3* and hopefully other targets. For our TRV delivery system, we generated an initial transgenic line expressing high levels of the base-editor; the line can be used repeatedly to create almost unlimited point mutations in the genome. We recognize that additional Cas9-expressing lines would be required to employ our approach in different genetic backgrounds or land races.

In conclusion, we developed a method to achieve heritable base-editing using a viral sgRNA delivery system in *Arabidopsis*, demonstrating loss-of-function mutations at *PDS3* and *CLA1* and gain-of-function mutations at *CESA3*. The system enables high-throughput analysis of gene function in planta in the M0 generation. Furthermore, homozygous mutant plants could be recovered in the M1. To date, our viral delivery method using TRV has been used to create heritable mutations in three species—*Arabidopsis*, *Nicotiana benthamiana*, and tomato (*Solanum lycopersicum*) (unpublished)—and probably can be expanded to other plant species. Indeed, recent work with *Barley Stripe Mosaic Virus* vectors demonstrated heritable editing through infection in wheat (*Triticum aestivum*) (Li et al., 2021). The simplicity, robustness, and versatility of our virus-induced base-editing method will open new avenues for accelerating both functional genomics and crop improvement.

## Supplemental data

The following materials are available in the online version of this article.

**Supplemental Figure S1.** TRV vectors, base-editor reagents, and the expression of the base-editor in transgenic *Arabidopsis* plants.

**Supplemental Figure S2.** Virus-mediated base-editing in *Arabidopsis* under a 24°C/22°C and 12-h/12-h day/night cycle.

**Supplemental Figure S3.** Pronounced *pds3* knockout phenotype at an early developmental stage in M0 *Arabidopsis* plants.

**Supplemental Figure S4.** Representative base-editing profiles in cauline leaves from plants grown at 18°C with a 16-h/8-h day/night cycle.

**Supplemental Figure S5.** Mosaic phenotypes of 8-day-old *PDS3* base-edited M1 seedlings.

**Supplemental Table S1.** The frequency of heritable base-editing at *PDS3* and *CLA1*.

**Supplemental Table S2.** The frequency of heritable base-editing at *CESA3*.

**Supplemental Table S3.** Primers used in this study.

**Supplemental Materials and Methods.**

## Supplementary Material

kiac206_Supplementary_Data

## Notes

D.F.V. conceived, designed, and supervised the project, and reviewed and edited the manuscript. D.L. conceived, designed, and performed experiments, analyzed the data, and wrote the manuscript. S.X., L.E.P., and L.I.D. performed the experiments. C.P. and Y.Q. generated the transgenic *Arabidopsis* lines expressing APOBEC3A (hA3A)/Y130F. U.N. and S.P.D.-K. provided the *A. tumefaciens* flooding infection protocol. E.E.E. provided the pEE083 vector. C.G.S. helped with experiments.

The author responsible for distribution of materials integral to the findings presented in this article in accordance with the policy described in the Instructions for Authors (https://academic.oup.com/plphys/pages/General-Instructions) is: Daniel F. Voytas (voytas@umn.edu).
