# Supplementary material for: Heritable base-editing in Arabidopsis using RNA viral vectors
Source: Plant Physiol. 2022 May 5;189(4):1920–4. doi: 10.1093/plphys/kiac206 (PMC9342971; doi:10.1093/plphys/kiac206)
Supplement: kiac206_Supplementary_Data [file kiac206_supplementary_data.zip › kiac206_Supplementary_Data/Supplemental Figures_R4_20220418.pptx]

## Slide 1
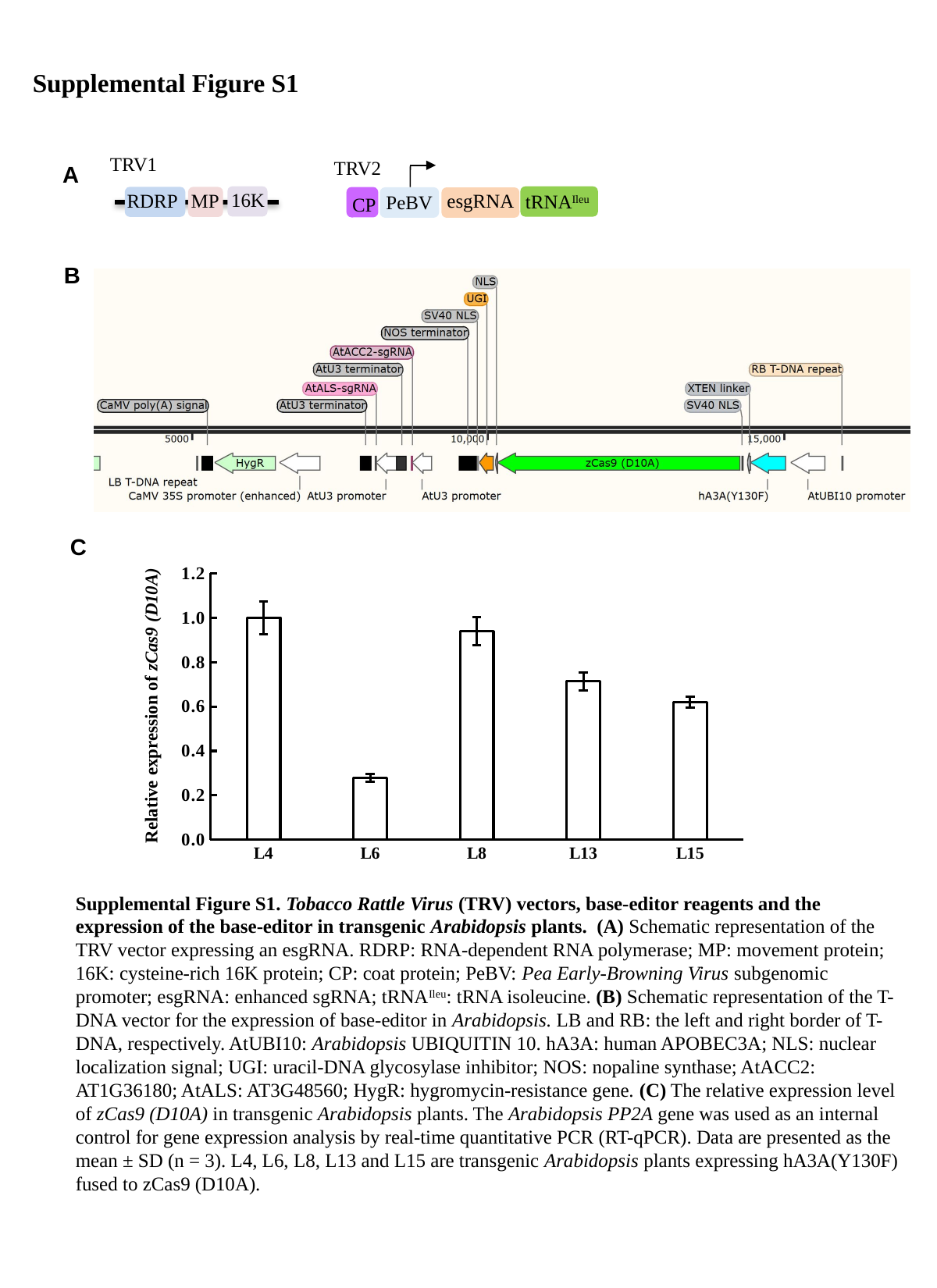

# Supplemental Figure S1
TRV1
16K
RDRP
MP
TRV2
A
esgRNA
tRNAIleu
PeBV
CP
B
C
### Chart
| Category | |
|---|---|
| L4 | 1.0 |
| L6 | 0.27844815809419254 |
| L8 | 0.9387865643053684 |
| L13 | 0.7130580937780248 |
| L15 | 0.6205103552886034 |Relative expression of zCas9 (D10A)
Supplemental Figure S1. Tobacco Rattle Virus (TRV) vectors, base-editor reagents and the expression of the base-editor in transgenic Arabidopsis plants. (A) Schematic representation of the TRV vector expressing an esgRNA. RDRP: RNA-dependent RNA polymerase; MP: movement protein; 16K: cysteine-rich 16K protein; CP: coat protein; PeBV: Pea Early-Browning Virus subgenomic promoter; esgRNA: enhanced sgRNA; tRNAIleu: tRNA isoleucine. (B) Schematic representation of the T-DNA vector for the expression of base-editor in Arabidopsis. LB and RB: the left and right border of T-DNA, respectively. AtUBI10: Arabidopsis UBIQUITIN 10. hA3A: human APOBEC3A; NLS: nuclear localization signal; UGI: uracil-DNA glycosylase inhibitor; NOS: nopaline synthase; AtACC2: AT1G36180; AtALS: AT3G48560; HygR: hygromycin-resistance gene. (C) The relative expression level of zCas9 (D10A) in transgenic Arabidopsis plants. The Arabidopsis PP2A gene was used as an internal control for gene expression analysis by real-time quantitative PCR (RT-qPCR). Data are presented as the mean ± SD (n = 3). L4, L6, L8, L13 and L15 are transgenic Arabidopsis plants expressing hA3A(Y130F) fused to zCas9 (D10A).

## Slide 2
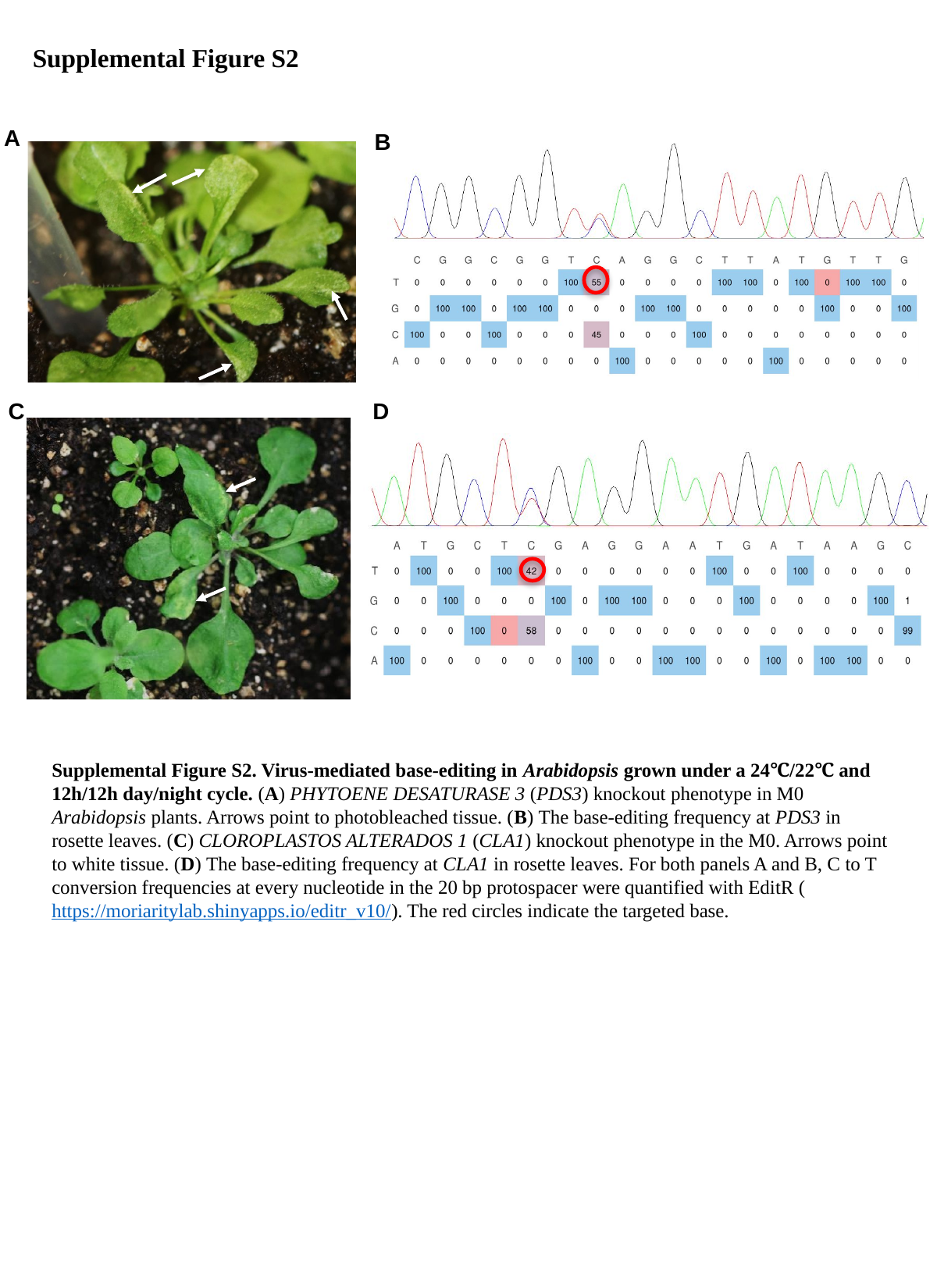

# Supplemental Figure S2
IMG_1917
A
B
C
D
Supplemental Figure S2. Virus-mediated base-editing in Arabidopsis grown under a 24℃/22℃ and 12h/12h day/night cycle. (A) PHYTOENE DESATURASE 3 (PDS3) knockout phenotype in M0 Arabidopsis plants. Arrows point to photobleached tissue. (B) The base-editing frequency at PDS3 in rosette leaves. (C) CLOROPLASTOS ALTERADOS 1 (CLA1) knockout phenotype in the M0. Arrows point to white tissue. (D) The base-editing frequency at CLA1 in rosette leaves. For both panels A and B, C to T conversion frequencies at every nucleotide in the 20 bp protospacer were quantified with EditR (https://moriaritylab.shinyapps.io/editr_v10/). The red circles indicate the targeted base.

## Slide 3
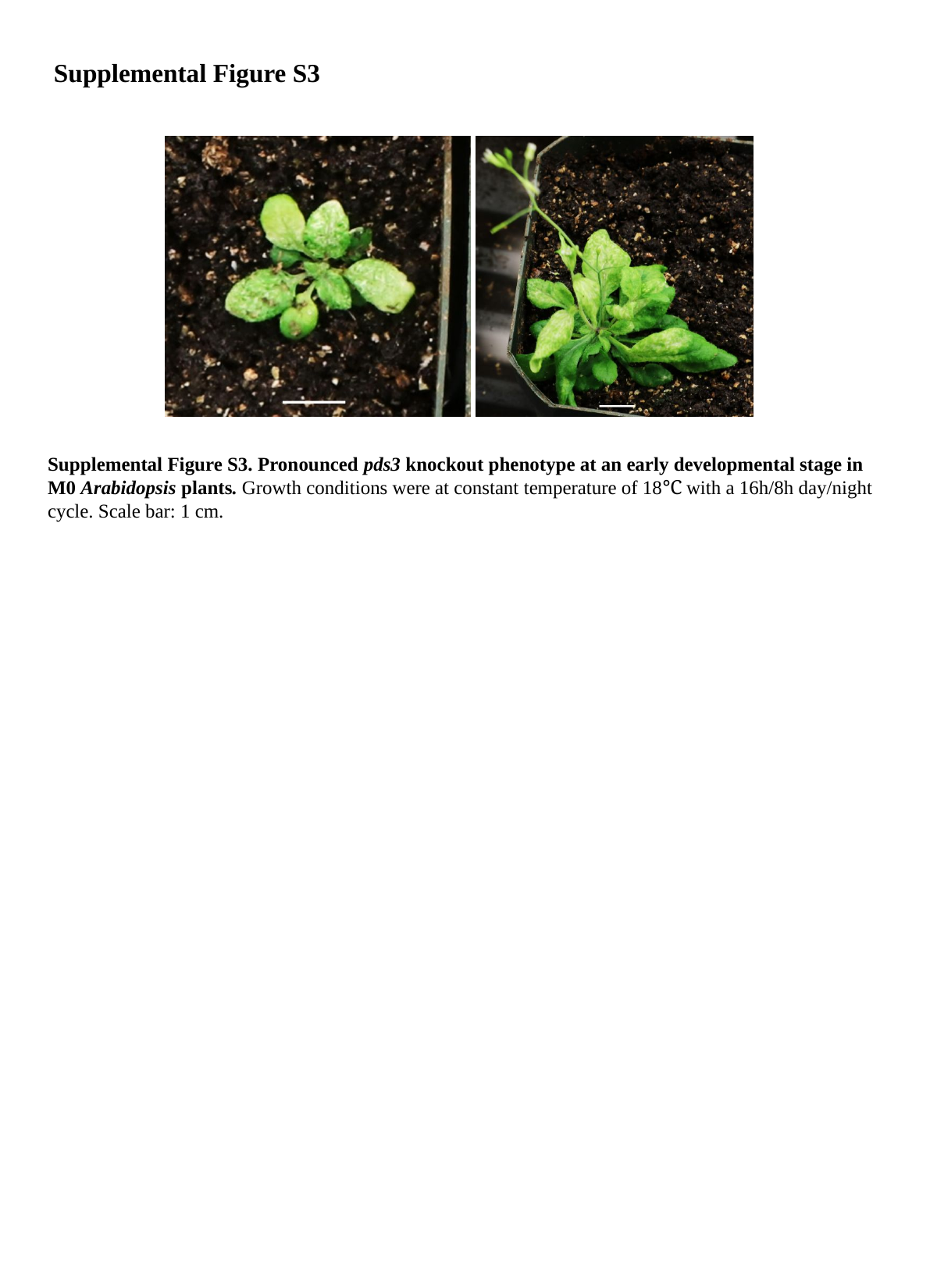

Supplemental Figure S3
Supplemental Figure S3. Pronounced pds3 knockout phenotype at an early developmental stage in M0 Arabidopsis plants. Growth conditions were at constant temperature of 18℃ with a 16h/8h day/night cycle. Scale bar: 1 cm.

## Slide 4
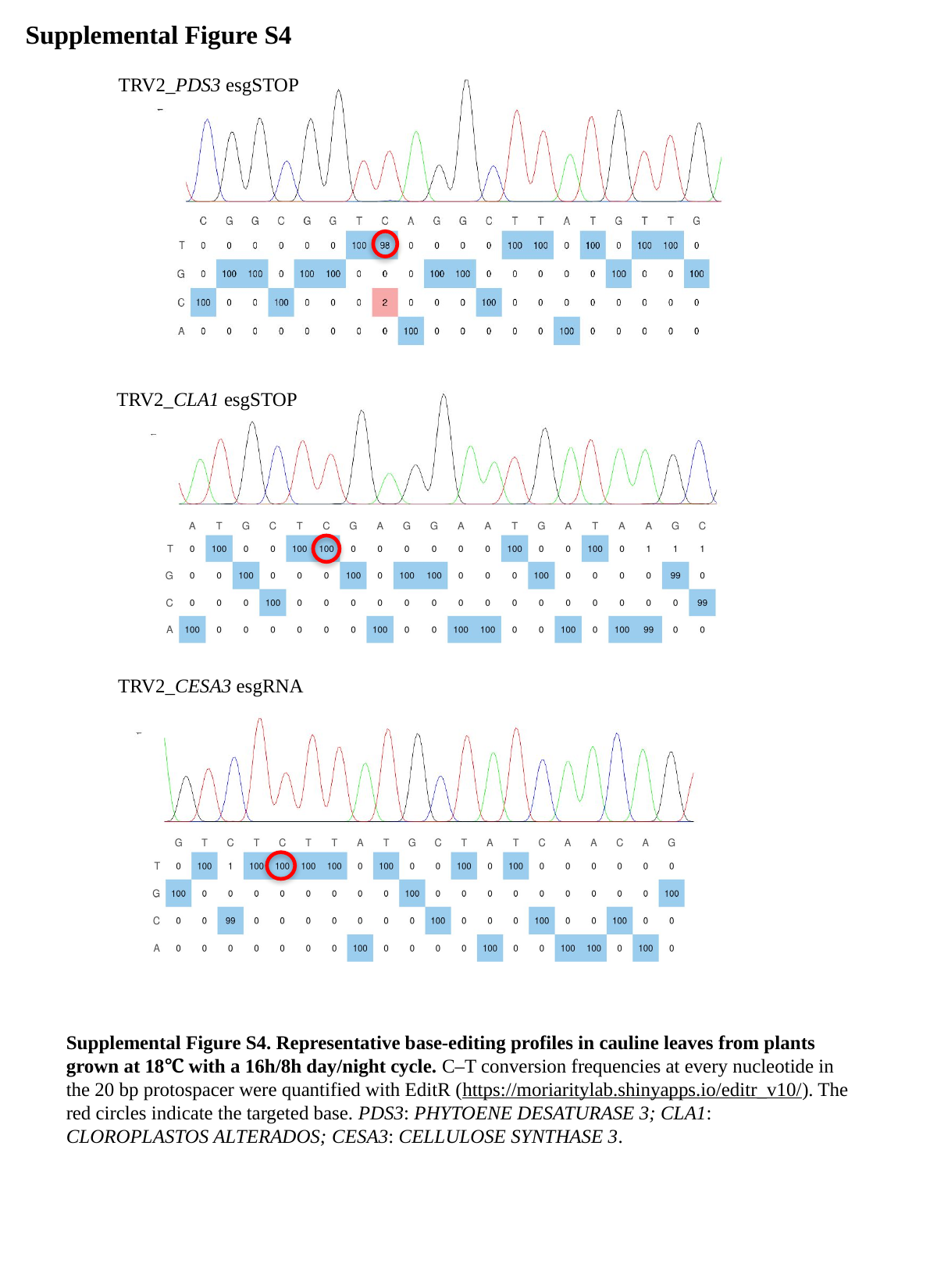

# Supplemental Figure S4
TRV2_PDS3 esgSTOP
TRV2_CLA1 esgSTOP
TRV2_CESA3 esgRNA
Supplemental Figure S4. Representative base-editing profiles in cauline leaves from plants grown at 18℃ with a 16h/8h day/night cycle. C–T conversion frequencies at every nucleotide in the 20 bp protospacer were quantified with EditR (https://moriaritylab.shinyapps.io/editr_v10/). The red circles indicate the targeted base. PDS3: PHYTOENE DESATURASE 3; CLA1: CLOROPLASTOS ALTERADOS; CESA3: CELLULOSE SYNTHASE 3.

## Slide 5
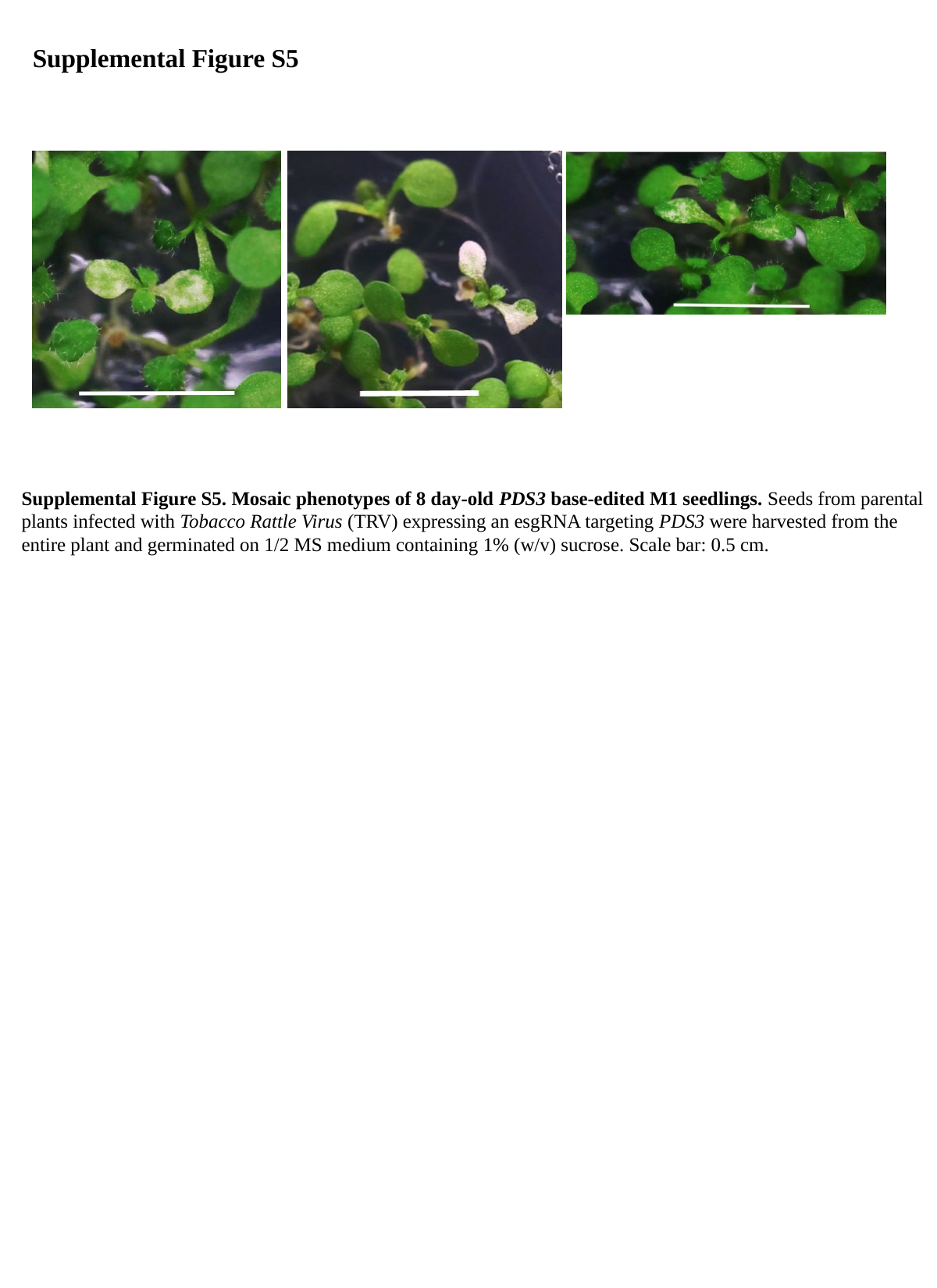

# Supplemental Figure S5
Supplemental Figure S5. Mosaic phenotypes of 8 day-old PDS3 base-edited M1 seedlings. Seeds from parental plants infected with Tobacco Rattle Virus (TRV) expressing an esgRNA targeting PDS3 were harvested from the entire plant and germinated on 1/2 MS medium containing 1% (w/v) sucrose. Scale bar: 0.5 cm.
