# Supplementary material for: Heritable base-editing in Arabidopsis using RNA viral vectors
Source: Plant Physiol. 2022 May 5;189(4):1920–4. doi: 10.1093/plphys/kiac206 (PMC9342971; doi:10.1093/plphys/kiac206)
Supplement: kiac206_Supplementary_Data [file kiac206_supplementary_data.zip › kiac206_Supplementary_Data/Supplemental Materials and Methods_R4_20220418.docx]

## **Materials and Methods**

## **Gene expression analysis**

The expression of human *APOBEC3A* (hA3A, Y130F)-zCas9(D10A) in transgenic *Arabidopsis* plants was analyzed by real-time quantitative PCR (RT-qPCR) as described by Liu et al. (2014). Total RNA was extracted from hygromycin-resistant *Arabidopsis* seedlings using the RNeasy Plant Mini Kit (QIAGEN, Cat. No. 74904). RNA reverse transcription and PCR amplification were performed with the Luna® Universal One-Step RT-qPCR Kit (New England Biolabs, Cat. No. E3005L). The *Arabidopsis PP2A* gene was used as an internal control. Specific primers designed for *PP2A* and *zCas9 (D10A)* were used in the qRT-PCR analysis (Supplemental Table S3). The relative gene expression level was quantified using the comparative CT method (Schmittgen and Livak, 2008).

## **TRV vector construction**

The enhanced sgRNA (Li et al., 2020) with an isoleucine tRNA (esgRNA-tRNA^Ileu^) was chemically synthesized by Integrated DNA Technology (Coralville, IA). With the esgRNA-tRNA^Ileu^ as a PCR amplification template, forward primers DL562, DL566 or DL567 and reverse primer DL423 were used to create the esgRNA-tRNA^Ileu^ targeting *CELLULOSE SYNTHASE 3* (CESA3), *CLOROPLASTOS ALTERADOS 1* (CLA1) or *PHYTOENE DESATURASE 3* (*PDS3*) (Supplemental Table S3). The PCR products were assembled into the TRV2 cloning vector pEE083 (vector sequences were uploaded to Addgene) (Ellison et al., 2020) using a Golden Gate Assembly cloning step. For TRV inoculation, the vectors were transferred into the *Agrobacterium tumefaciens* strain, GV3101, using the freeze-thaw method (Höfgen and Willmitzer, 1988).

## **TRV inoculation**

TRV inoculation was performed using the Agro-flooding method (Nagalakshmi et al., 2022). *Arabidopsis* seedlings were germinated on 1/2 MS medium. Two *A. tumefaciens* strains were mixed in a 1:1 ratio and adjusted to OD_600_ =1.5. One strain harbors a T-DNA with TRV1 (pNJB069) and the other harbors TRV2 with the esgRNA-tRNA^Ileu^. The *A. tumefaciens* suspension was dispensed directly into a Petri dish with 6 to 7 day-old seedlings. After 3 days of co-culture, seedlings were transplanted to soil and maintained in a growth chamber.

## **Optimized plant growth conditions for TRV-mediated base-editing**

The *Arabidopsis* plants infected with TRV were grown in a growth chamber at the following conditions: a constant temperature of 18℃; a 16h/8-h day/night cycle; a light intensity of 100 umol m^-2^ s^-1^; 50% humidity.

## **Analysis of somatic editing efficiencies**

Genomic DNA was extracted from leaves of TRV infected plants according to the instructions provided with the DNeasy Plant Mini Kit (QIAGEN, Cat. No. 69106). Target sites were amplified by PCR using genomic DNA as a template with gene-specific primers (Supplemental Table S3). The PCR products were subjected to Sanger sequencing. Base-editing frequencies at every nucleotide in the 20 bp protospacer were quantified using EditR (<https://moriaritylab.shinyapps.io/editr_v10/>) (Kluesner et al., 2018).

## **Analysis of heritable editing efficiencies**

All seeds were harvested from plants infected with TRV. For the *PDS3* and *CLA1* experiments, seeds were germinated on 1/2 MS medium containing 1% (w/v) sucrose. For the *CESA3* experiment, seeds were plated on 1/2 MS medium containing 1% (w/v) sucrose and 1 μM C17 (ChemBridge, catalog no. 7693622). At least two leaves from each seedling were collected, pooled and used for genomic DNA extraction. Methods of PCR amplification, Sanger sequencing and base-editing analyses were the same as described above for measuring somatic editing efficiencies. The following definitions were used to describe genotypes in the *PDS3* and *CLA1* experiments: white seedlings with the same mutations in both alleles, homozygous mutants; green seedlings with white or yellow sectors with evidence of target gene editing, mosaic mutants; green seedlings with editing frequencies between 15.1%–35.0% or 65.1%–85.0%, mosaic mutants; green seedlings with editing frequencies between 35.1%–65.0%, heterozygous (monoallelic) mutants; green seedlings with editing frequencies between 0%–15.0%, wild-type. For the *CESA3* experiment, C17-tolerant seedlings with the same mutations in both alleles and the editing frequencies between 85.1%–100.0% were defined as homozygous.

# **References**

**Ellison EE, Nagalakshmi U, Gamo ME, Huang P-j, Dinesh-Kumar S, Voytas DF** (2020) Multiplexed heritable gene editing using RNA viruses and mobile single guide RNAs. Nat Plants **6:** 620-624

**Höfgen R, Willmitzer L** (1988) Storage of competent cells for *Agrobacterium* transformation. Nucleic Acids Res **16:** 9877

**Kluesner MG, Nedveck DA, Lahr WS, Garbe JR, Abrahante JE, Webber BR, Moriarity BS** (2018) EditR: a method to quantify base editing from Sanger sequencing. CRISPR J **1:** 239-250

**Li C, Zong Y, Jin S, Zhu H, Lin D, Li S, Qiu J-L, Wang Y, Gao C** (2020) SWISS: multiplexed orthogonal genome editing in plants with a Cas9 nickase and engineered CRISPR RNA scaffolds. Genome Biol **21:** 1-15

**Liu D, He S, Zhai H, Wang L, Zhao Y, Wang B, Li R, Liu Q** (2014) Overexpression of *IbP5CR* enhances salt tolerance in transgenic sweetpotato. Plant Cell, Tissue Organ Cult **117:** 1-16

**Nagalakshmi U, Meier N, Liu J-Y, Voytas DF, Dinesh-Kumar SP** (2022) High-efficiency multiplex biallelic heritable editing in *Arabidopsis* using an RNA virus. Plant Physiology

**Schmittgen TD, Livak KJ** (2008) Analyzing real-time PCR data by the comparative CT method. Nat Protoc **3:** 1101-1108
