# Supplementary material for: Heritable base-editing in Arabidopsis using RNA viral vectors
Source: Plant Physiol. 2022 May 5;189(4):1920–4. doi: 10.1093/plphys/kiac206 (PMC9342971; doi:10.1093/plphys/kiac206)
Supplement: kiac206_Supplementary_Data [file kiac206_supplementary_data.zip › kiac206_Supplementary_Data/Supplemental Tables_R4_20220418.docx]

**Supplemental Tables**

**Supplemental Table S1. The frequency of heritable base-editing at *PDS3* and *CLA1.***

| **Target_ Parental line** | **White seedlings (Homozygous)** | **Green seedlings with white or yellow zones (mosaic)** | **Green seedlings** | | **Total frequency** |
| --- | --- | --- | --- | --- | --- |
|  |  |  | **Hetero-zygous** | **Mosaic** |  |
| *PDS3*_P1 | 2/171, 1.17% | 3/171 (C to T); 1/171 (-14bp) | 0/61 | 0/61 | 3.51% |
| *PDS3*_P2 | 2/339 (-4bp/-4bp), 0.59% | 1/339 | 1/32 (C to T); 1/32 (-4bp/WT) | 0/32 | 7.08% |
| *PDS3*_P3 | 21/236, 8.90% | 0/236 | 7/38 | 0/38 | 25.68% |
| *CLA1*_P1 | 2/138, 1.45% | 2/138 | 1/62 | 0/62 | 4.46% |
| *CLA1*_P2 | 1/234, 0.43% | 5/234 | 2/53 | 1/53 | 8.08% |
| *CLA1*_P3 | 1/198, 0.51% | 2/198 | 2/67 | 0/67 | 4.45% |

Note: All seeds were harvested from plants infected with TRV vectors expressing esgRNAs. Seeds were germinated on 1/2 MS medium containing 1% (w/v) sucrose. All white seedlings and green seedlings with white or yellow sectors were genotyped. Green seedlings were randomly collected and genotyped. One green seedling with white sectors from *PDS3*_P1 had a 14bp deletion; two white seedlings and one green seedling from *PDS3*_P2 had 4bp deletions. All other mutations were C-to-T transitions. Total frequency = no. of white seedlings (homozygous)/no. of total germinated seedlings + no. of green seedlings with white or yellow zones (mosaics)/no. of total germinated seedlings + ((no. of green heterozygous seedlings and mosaics/no. of randomly genotyped green seedlings) X (no. of total germinated seedlings - no. of white seedlings (homozygous) - no. of green seedlings with white or yellow zones (mosaic)))/no. of total germinated seedlings.

**Supplemental Table S2. The frequency of heritable base-editing at *CESA3.***

| **Target_Parental line** | **Screened progeny** | **Homo-zygous** | **Hetero-zygous** | **Mosaic** | **Total frequency** |
| --- | --- | --- | --- | --- | --- |
| *CESA3*_P1 | 250 | 5, 2% | 0, 0% | 2, 0.8% | 2.8% |
| *CESA3*_P2 | 335 | 7, 2.09% | 1, 0.3% | 0, 0% | 2.39% |
| *CESA3*_P3 | 285 | 4, 1.4% | 2 (C-to-T) 1(C-to-G), 1.05% | 1 (C-to-T and C-to- G), 0.35% | 2.81% |

Note: All seeds were harvested from plants infected with TRV vectors expressing esgRNAs. Seeds were germinated on 1/2 MS medium supplemented with 1 μM C17. C17- tolerant seedlings were genotyped. C-to-G editing was observed in two seedlings from *CESA3_*P3; all other mutations were C-to-T transitions.

**Supplemental Table S3. Primers used in this study.**

| **Primer name** | **Primer sequence (5′-3′)** |
| --- | --- |
| *PP2A*_qRT_F | AACCGCTTGGTCGACTATCG |
| *PP2A*_qRT_R | AACGTGGCCAAAATGATGC |
| zCas9(D10A)_qRT_F | CCTCGTGGAGGAGGATAAGA |
| zCas9(D10A)_qRT_R | AGATGGTAGATCGTAGGGTACTT |
| DL562_*CESA3*_TRV2_F | ACGTCTCGCAGGCACCTGCGCTCAAACGTCTCTTATGCTATCAACAGGTTTAAGAGCTATGCTGGAAACAGCA |
| DL566_*CLA1*_TRV2_F | ACGTCTCGCAGGCACCTGCGCTCAAACATGCTCGAGGAATGATAAGCGTTTAAGAGCTATGCTGGAAACAGCA |
| DL567_*PDS3*_TRV2_F | ACGTCTCGCAGGCACCTGCGCTCAAACCGGCGGTCAGGCTTATGTTGGTTTAAGAGCTATGCTGGAAACAGCA |
| DL423_TRV2_R | TCGTCTCCCGAGCACCTGCTAGTCACTTGCTTCCGGCGGGGCTCG |
| *CESA3*_genotyping_F | GACTGGAGTTCAGACGTGTGCTCTTCCGATCTCCTCAAAGTCCTTGCCGGTATTG |
| *CESA3*_genotyping_R | ACACTCTTTCCCTACACGACGCTCTTCCGATCTGTAGGAGTCCGGTTCTGTCGA |
| *CLA1*_genotyping_F | GACTGGAGTTCAGACGTGTGCTCTTCCGATCTGAATACTGGTCTTTATGGACTTGATG |
| *CLA1*_genotyping_R | ACACTCTTTCCCTACACGACGCTCTTCCGATCTTGGTTCTGGTACTCTTAACTTCTTTAAG |
| *PDS3*_genotyping_F | GACTGGAGTTCAGACGTGTGCTCTTCCGATCTGTTGTTGCTGTTGGATTTACG |
| *PDS3*_genotyping_R | ACACTCTTTCCCTACACGACGCTCTTCCGATCTTGATTCAGCATGCTAACTACTCTA |
